# Amnion bilayer for dressing and graft replacement for delayed grafting of full-thickness burns; A study in a rat model

**DOI:** 10.1371/journal.pone.0262007

**Published:** 2022-01-21

**Authors:** Normalina Sandora, Nur Amalina Fitria, Tyas Rahmah Kusuma, Gammaditya Adhibarata Winarno, Sanjaya Faisal Tanjunga, Aditya Wardhana

**Affiliations:** 1 Faculty of Medicine, Universitas Riau, Pekanbaru, Indonesia; 2 Indonesian Medical Education and Research Institute (IMERI), Jakarta, Indonesia; 3 Burn Unit, Cipto Mangunkusumo National General Hospital, Jakarta, Indonesia; 4 Department of Surgery, Plastic and Reconstructive Surgery Division, Cipto Mangunkusumo National General Hospital, Jakarta, Indonesia; 5 Faculty of Medicine, Universitas Indonesia, Jakarta, Indonesia; University College London Institute of Child Health, UNITED KINGDOM

## Abstract

Burn is a common case in developing countries, with over half of fire-related deaths reported in Southeast Asia and full-thickness burns as a high mortality risk. Human amnion has been used as a wound dressing for centuries. In this study, a decellularised amnion overlaid with fibrin, “amnion bilayer (AB),” was used as a dressing immediately after burn and as a graft to replace the scar in Sprague-Dawley rats subjected to full-thickness burn model. The aim was to observe whether amnion bilayer can reduce damages in third-grade burn when skin replacement is deemed impossible. The burn was induced using an electrical solder, heated for 5 mins, and contacted on the rat’s bare skin for 20 s. AB was applied as a (i) dressing immediately after induction and graft after eschar removal. Two groups (n = 6) were compared: AB and Sofra-Tulle ^®^, the National Hospital of Indonesia (NHI) protocol. Sections were stained with hematoxylin and eosin and Masson trichrome stains. Immunohistochemistry labelling was used to indicate scars (α-smooth muscle actin [α-SMA] and collagen-1) and angiogenesis (von Willebrand factor). Also, the macrophages inflammatory protein-3α (MIP-3α) indicates an early inflammatory process. The post dressing of the AB group demonstrated hair follicle remains and adipose tissue development. The NHI group appeared with a denatured matrix. Complete healing was seen in the AB group after 28 days with skin appendages similar to normal, while the NHI group showed no appendages in the centre of the actively inflamed area. The α-SMA was found in both groups. Collagen-1 was highly expressed in the NHI group, which led to a scar. Angiogenesis was found more in the AB group. The AB group had shown the capacity to accelerate complete healing and recover skin appendages better than the current protocol.

## Introduction

Burns are primarily found in developing countries, and half of the incidents were reported in Southeast Asia [1]. The World Health Organization database in 2002 showed that the mortality rate due to fire causes were 11.6 deaths per 100,000 population per year in Southeast Asia [2]. The mortality rate is linear to the total body surface area (TBSA) exposed to the wound, which is lower when it involves less than 10% of the TBSA [3], but not when more than 50% [4].

According to the depth of destruction, there are three grades of burn: superficial (grade 1), partial (grade 2A) or deep partial-thickness (grade 2B), and full-thickness (grade 3) burns [5]. Grade 1 superficial is the mildest type with minor capillary destruction [6, 7]. The partial/deep partial-thickness burns have the epidermis detached from the dermis [7, 8]. In contrast, full-thickness burns have distinct damages of the epidermal layer, skin appendages destruction until the panniculus carnosus muscle [9, 10].

In general, wound healing involves several stages: inflammation, granulation tissue formation, re-epithelialisation, and remodelling. This complex process is regulated by the complex signalling molecule involving numerous growth factors, cytokines and chemokines [11]. Cytokines are small proteins secreted by cells and have a specific effect on cell interactions. Cytokines consists of several types included lymphokine (cytokines produced by lymphocytes), monokine (cytokines produced by monocytes), chemokine (cytokines with chemotactic activities), and interleukin (cytokines produced by one leukocyte and acting on other leukocytes). Cytokines in the wound healing process involve both pro and anti-inflammatory [12].

A full-thickness burn needs surgical debridement to remove the eschars to prevent infection and optimise treatment [13]. Patients with severe conditions need to postpone eschar-removal surgery until the general condition has improved. The debridement will need immediate replacement using a skin graft [14]. Nonetheless, skin graft, whether autologous, allogeneic or other substitutes, is still yet ideal. Even though the autologous source is a gold standard; however, it is too much for elderly patients to undergo multiple procedures, limited source, while the allogeneic source should deal with immunocompatibility issues. Therefore, studies searching for skin substitutes from various biomaterials that mimic natural skin are rigorously conducted worldwide [15].

In this study, the amnion bilayer (AB) was applied as a dressing immediately after burn induction, followed by AB as graft replacement started from Day 7 after scar removal. AB is a decellularised human amnion membrane (hAM) overlaid with fibrin. The hAM naturally has anti-inflammatory and antibacterial capacities [16]. The hAM is a waste product in caesarean section procedures and normal labour; therefore, it has no ethical objections. The amnion used in this study was initially removed from the donor cells through a decellularisation process. The decellularised amnion was then overlaid with fibrin. Fibrin was isolated from the whole blood, following a previous study [16]. Fibrin contains many growth factors such as platelet-derived growth factor (PDGF), transforming growth factor-beta (TGF-β), and vascular endothelial growth factor (VEGF). Incomplete healing leads to scar formation. The activation of basic fibroblast growth factor (bFGF) inhibitor and PDGF prevents this process [10, 17, 18]. Therefore, a combination of a decellularised amnion membrane with fibrin was purposed to enhance the wound-healing process.

This study aimed to investigate the application of AB as a dressing for full-thickness burns in rat models. In a scenario where health care facilities are insufficient, and an eschar removal is yet possible, a dressing is needed to allow transportation to the higher facility. AB was investigated for its capacity to reduce the damages when applied immediately after severe burn until skin transplantation is available.

## Materials and methods

According to the Guide for Care and Use of Laboratory Animals of the National Institutes of Health. The ethical board of the Faculty of Medicine, University of Indonesia (UI), had approved all procedures on this study (Protocol Number: 19-08-1041; Ethical approval number: KET-994/UN2.F1/ETIK/PPM.00.02/2019). The methods to induce burn wounds were done following a previous study [19]. Patients for amnion membrane donation had been adequately informed about the study, and the consent forms were signed.

### Animals

#### Rat handling

Rat as burn model has been well established in the literature [20]. Eighteen male Sprague-Dawley albino rats aged 2–3 months and weighed 200–350 g were used as the animal model. The rats were obtained from the animal facility of the Faculty of Medicine-University of Indonesia. They acclimated for seven days before the experiment; six were placed in each cage, kept stable at 25°C, ventilated with a closed-air system, exposed to light 12 h per day, and allowed access to food and water *ad libitum*. Three groups were compared. Group 1 was for the no treatment, Group 2 was treated with AB, and Group 3 to the standard protocol applied in the National Hospital of Indonesia (NHI); Sofra-Tulle^®^, as a dressing to full-thickness burn patients when skin grafts are impossible. All interventions such as burn induction, graft dressing and transplantation, or termination were performed under general anaesthesia. The burn induction method was done following a previous study [19].

On the first day of the intervention, rats were weighed, then anaesthetised with intramuscular injection of a combination of 100 mg/kg body weight ketamine and 10 mg/kg xylazine (Interchemie, Holland) [21]. After 5 minutes post-injection, a pinch test was performed to indicate adequate anaesthesia had been achieved [22]. If the rats were still responsive, an additional ketamine/xylazine injection of half the initial dose was added [23]. On average, it took two minutes for the animals to lose their reflex after ketamine+ xylazine injection. The duration of anaesthesia was never less than 60 mins but not longer than 80 mins. Most interventions in our study were performed in less than an hour.

#### Methods of sacrifice (euthanasia)

The Methods of euthanasia followed the AVMA Guidelines on Euthanasia, ACLAM Task Force Guidelines on Euthanasia [24]. Chemical anaesthetic overdose was chosen for euthanasia; the standard 2–3 times anaesthetic dose was used for euthanasia. Once injected, further cervical dislocation was applied to ensure the animals were euthanised. Other suggestions such as exsanguination or thoracotomy can also assure death [24, 25]. In this study, ketamine–xylazine was used both for anaesthesia and euthanasia. The ratio of ketamine/ xylazine was calculated accurately to prevent the animals model from suffering.

## Graft preparation and microbiological tests

The hAM was obtained from caesarean section procedures. The donor was nonreactive to human immunodeficiency virus (HIV) and hepatitis B and C. The hAM then separated from the chorion, washed consecutively using Triton-X (0.1%; w/v) and sodium dodecyl sulphate (0.05%; w/v), coated by fibrin, and then incubated at 37°C for 24 h. All grafts were randomly checked for microbiological tests (sterility assays). The test used tryptone soy broth (Sigma, USA), Sabouraud dextrose agar (Sigma, USA), and blood agar (Merck, Germany) to verify the biocompatibility towards humans bone-marrow MSC and acellularity using H&E (Scytek, USA) and DAPI staining (Abcam, USA). All grafts were found acellular, had no growth, and were biocompatible.

### Induction of a full-thickness burn in rats and wound size measurement

Once confirmed anaesthetised, the rats were induced for burn wound, followed by the previous study [19, 26]. Briefly, rats were placed with the dorsomedial area exposed, shaved clean, and then contacted lightly with an electrical solder (2 × 4 cm^2^) for 20 seconds. The electrical solder was initially heated for 5 minutes. The eschar size was measured, and then AB was applied as a dressing (n = 6) for seven days, covered with gauze, and suture to keep the AB in a place. The NHI group was treated similarly to the AB group, however Sofra-Tulle ^®^ was used as the dressing. The eschar was removed on Day 7 in both groups, replaced with AB or Sofra-Tulle ^®^, respectively. The termination was conducted on all rats once a rat had been shown to recover fully. Throughout the study, records of rats’ behaviour, weight, and wound size were observed every day. The wound-size reduction was calculated using the formula below, as published by Chen et al., 2008 [27]:

$$\frac{Area\;of\;the\;wound\;on\;Day\;0-Area\;of\;the\;wound\;of\;the\;measuring\;day\;x\;100\%}{Area\;of\;the\;wound\;on\;Day\;0}$$

### Histological analysis and immunohistochemical labelling

For histology, all specimens were fixed in 10% (v/v) neutral-buffered formalin solution (Leica, USA) for 48 h, processed automatically in a tissue processor (Leica, USA), paraffin blocked, and sectioned by 5 μm thickness. Sections were stained using hematoxylin and eosin (H&E; Scytek, USA) and Masson trichrome stains (Scytek, USA) according to the manufacturer’s instructions, visualised using the brightfield microscope, and captured using Zeiss Axio Imager (Zeiss, Germany).

All immunohistochemical labelling followed the biotin-avidin labelling protocol against several markers. The collagen-1 (1:100 dilution, Invitrogen, USA) and α-smooth muscle actin (α-SMA; 1:400 dilution, Sigma) indicated the scar formation. The Von Willebrand factor (vWF; 1:200 dilution, Dako, Denmark) indicates angiogenesis (22). The macrophages antigen (macrophage inflammatory protein-3-α/ MIP-3-α; 1:500, Abcam, USA) show an early inflammatory process. Antigen retrieval was performed using heat-induced epitope retrieval with 10 mM citric acid pH 6 (microwave at 95–100°C, 10 min; α-SMA), Tris-EDTA pH 9 (microwave at 95–100°C, 10 min; MIP-3-α) and the enzymatic reaction using proteinase K (Dako, Denmark) at 25°C for 10 min (collagen-1 and vWF).

### RT-qPCR

All specimens were cut into 5x5x5 mm^3^ and stored in RNAlater solution (ThermoFisher, USA) until all samples were collected. The RNA was isolated using TRIzol™ Reagent (ThermoFisher, USA) and converted into complementary DNA using SensiFAST™ cDNA Synthesis Kit (Bioline, USA). The gene expressions were identified using the real-time qPCR method using SensiFAST™ SYBR Lo-ROX Kit (Bioline, USA) and performed with the AppliedBioscience-7500 Fast Dx Real System PCR instruments (ThermoFisher, USA). The cytokines to indicate inflammation, such as TNFα, IL-6, MMP-1, and anti-inflammatory IL-10, were identified. The primer sequence used in this study are IL-6 (F 5’CTGGTCTTCTGGAGTTCCGT3’, R 5’TGCTCTGAATGACTCTGGCT3’), IL-10 (F 5’TGATGCCCCAAGCTGAGAAC3’, R 5’AATCGATGACAGCGCCGTAG3’), MMP-1 (F 5’AGTCTGGGTTGTTTCGAGAGC3’, R 5’GGCAGCGTCAATGTGTGTCA3’), TNF-α (F 5’TCAGAAACACACGAGACGCT3’, R 5’CATTGGAATCCTTGCCGGTG3’), and GADPH as the reference gene (F 5’TCTCTGCTCCTCCCTGTTC3’, R 5’ACACCGACCTTCACCATCT3’). All PCR data were calculated using Livak analysis.

### Data analysis

The images of the burn wound were documented daily using a digital camera (Canon, Japan), captured from a consistent height and distance to the object, and a ruler was placed next to the wound and used as a reference pixel scaling while processing the image in ImageJ software (imageJ.NHI.gov). The method to analyse wound area was done following a previous study [28]. The data were then processed in Excel 2016 (Microsoft, USA and presented as means ± 95% C.l). Differences among groups were highlighted using a one-way ANOVA, followed by posthoc analysis using GraphPad Prism6 (GraphPad, USA). A p-value less than 0.05 was considered significant.

## Results

**Fig 1:**
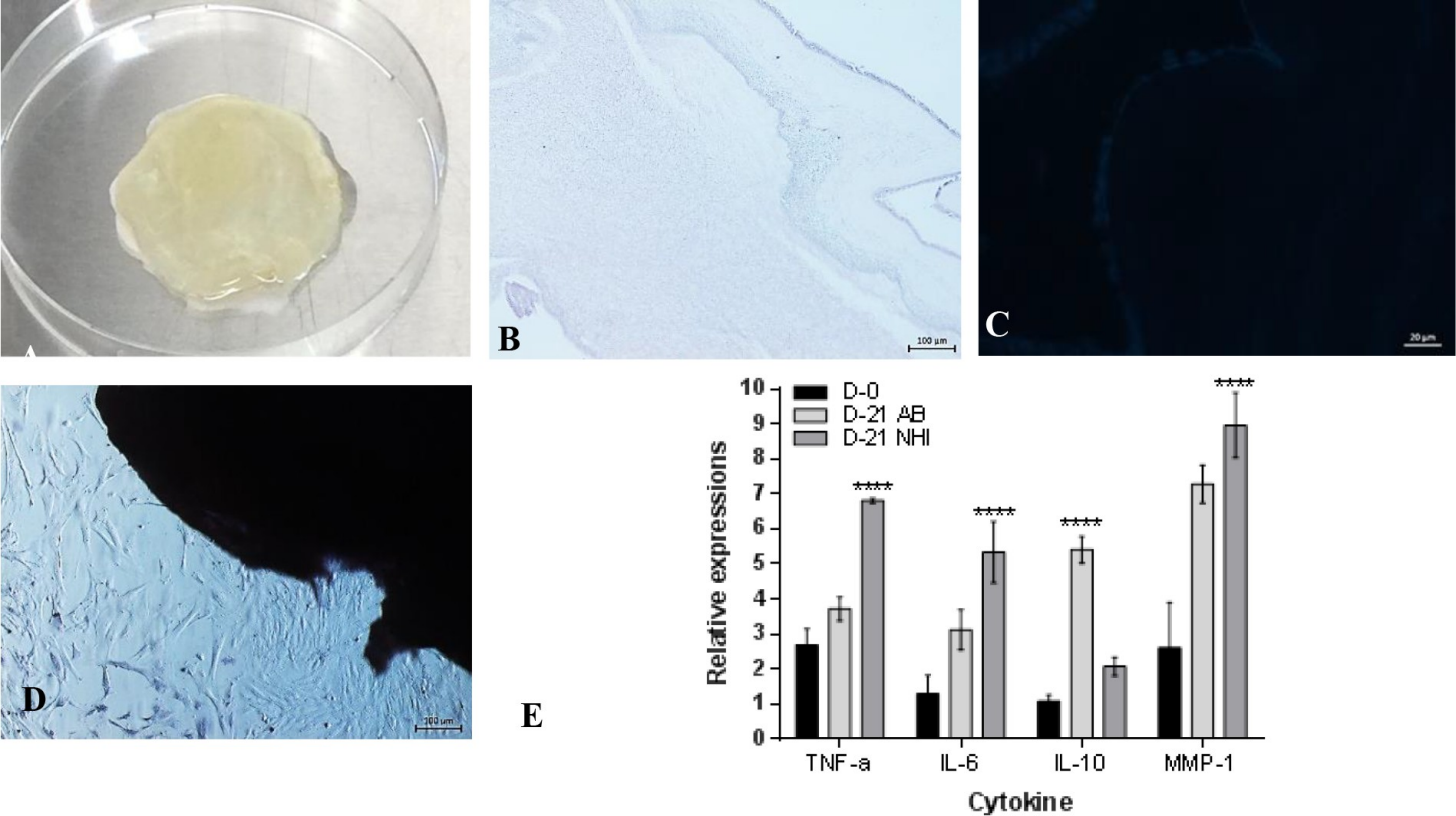
Amnion bilayer graft. (A) macroscopic. The sections after (B) H&E staining (100x mag), (C) DAPI staining (400x mag). (D) Contact toxicity towards human BM-MSC (100 mags), (E) RT-PCR indicated cytokine expressions of the explant of the burn wound.

The amnion bilayer (Fig 1A) prepared for this study was acellular indicated by Fig 1B and 1C. It showed to be biocompatible (Fig 1D) as the cells were attached to the graft, with cellular colony had similar morphology to the cell culture. The biomechanical properties determined using Dynatron showed that the ultimate tensile stress (UTS) of the AB was 0.42±0.15 MPa, Ultimate tensile strain (UTStrain) was 0.63±0.26MPa, and Young modulus was 0.69±0.37MPa.

**Fig 2:**
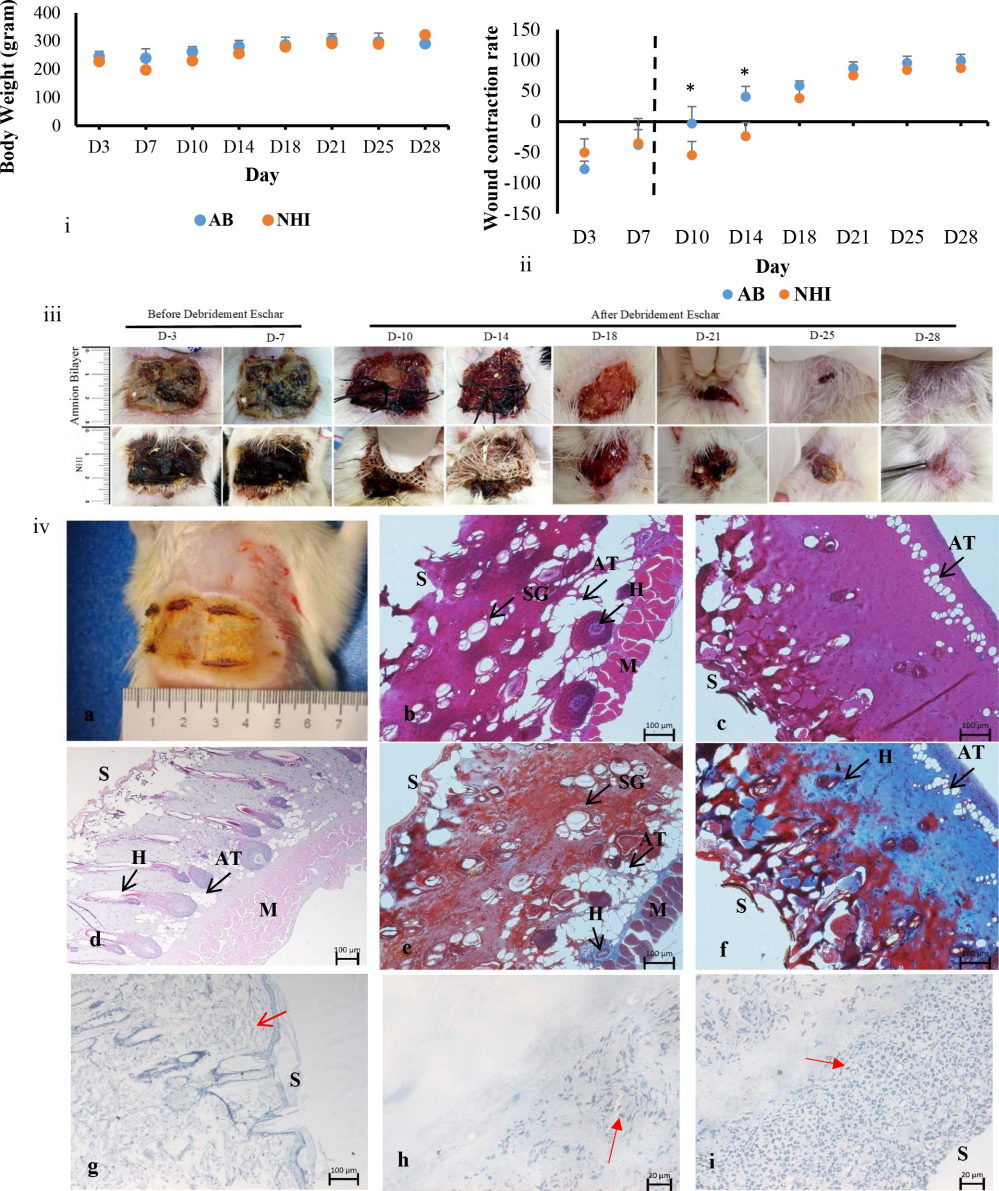
Full-thickness burn model in rats. Intervention up to Day-7 was for dressing, and from Day-10 onwards were the eschar removal and transplantation. (i) Records of rats’ body weight over time. (-ii-) Wound contraction rate. Data are expressed as means (n = 6) ± SD,*p<0.05. (iii) Images of rats wound treatment with AB (top row) and NHI (bottom). (iv) (a) immediately after burn induction, and (d) the H&E staining of the post-induction. (b,e) H&E and Masson staining of the AB dressing, (c, f) H&E and Masson staining of the Sofra-Tulle ^®^, (g) MIP-3 α staining of normal skin rat, (h) MIP-3 α staining of AB dressing and (i) MIP-3 α staining of the Sofra-Tulle ^®^. Images of H&E and Masson were presented at 100x magnification. Images of MIP-3 α was presented at 400x magnification—S = Surface, H: hair follicles, AT Adipocyte tissue, SG: sebaceous gland, M = Muscle.

A rectangular eschar formed immediately after heat induction (Fig 1A). The rat’s body weight and wound size were recorded on the first visit after intervention (p > 0.05; Day 3; Fig 2i and 2ii).

One day after intervention, AB was found to adhere tightly to the wound, merge to the eschar, and develop a dried crust by Day 3. While in the NHI group, the gauze was found stuck to the wound, formed a dark and wet necrotic crust by Day 3, which remained in place until Day 7. The wound contraction was significantly higher in the AB group than in the NHI group; by Day 10 (up to 5%) and Day 14 (41.20%; p < 0.05; Fig 2ii). Wound size among the AB group healed rapidly from Day 18 until the wound size was less than 5% by Day 25, and recovered completely without scars after Day 28 (p > 0.05), with hair had grown and covered the post-wound areas. The wound of the NHI group started to heal faster after Day 18, achieved up to 60–70% reduction by Day 21, similar to the AB group (p > 0.05), and 80% on Day 25 (p > 0.05). The wound size of the NHI group had reduced up to 85% on Day 28; meanwhile, the AB group had recovered.

Once induced (Fig 2iv a), the H&E staining of the burn wound showed severe third-grade burn with massive destruction of the dermis, damaged appendages, nearly exposed the muscle, while the epidermis had disappeared (Fig 2iv d). After seven days of dressing treatment, the H&E and Masson staining of AB group sections started to develop several skin appendages such as sebaceous glands and hair follicles, and adipose tissues were also found scattered all over the dermis. However, the ECM degradation was still dominant (Fig 2iv b and e). In contrast, the NHI group appeared with severe homogeneous denatured collagen-extracellular matrix (ECM) stained in deep primarily red at the surface, with irregular pyknotic nuclei indicating dead cells, The granulation tissues appeared blue by Masson staining, dominated the deeper layer (Fig 2iv c and f). The MIP-3 α labelling the sections after seven days of dressing in the NHI group (Fig 2iv and 2i) was massive compared to the AB group (Fig 2iv h).

**Fig 3:**
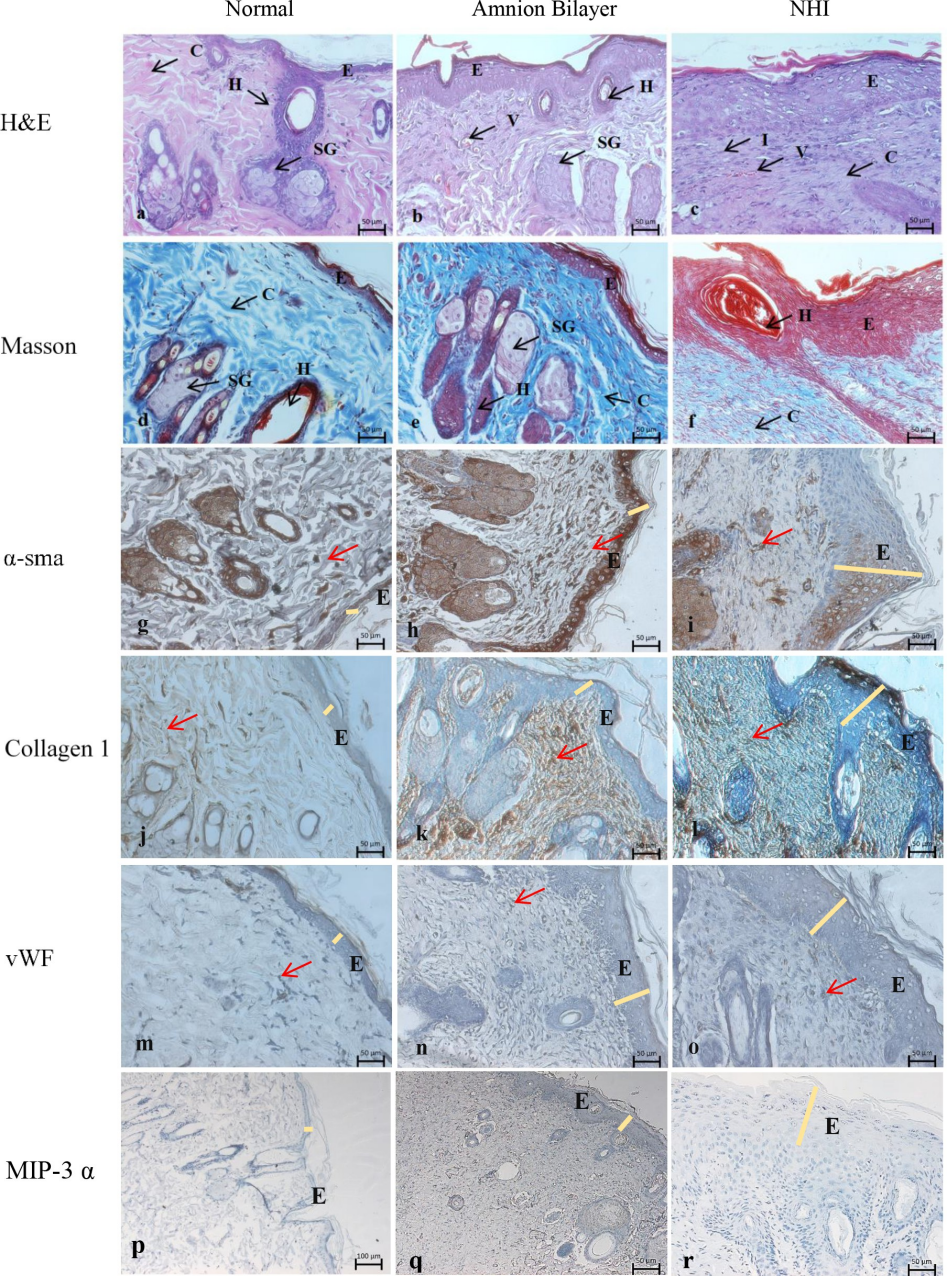
Histological sections of the wound after Day-28. (a, d, g, j, m, p) Normal rat skin; (b, e, h, k, n, q) The AB, and (c, f, i, l, o, r) the NHI group. [29] (a-c) H&E and (d-f) Masson staining. IHC labelling against (g-i) α-SMA, (j-l) Collagen-1, (m-o) vWF, (p-r) MIP-3 α. E: epidermis, H: hair follicles, AT Adipocyte tissue, SG: sebaceous gland. All images were presented at 200x magnification, the red arrow points to the interested cells, and the yellow line indicates the epidermis thickness.

In the AB group, the skin tissue that developed after the wound had healed entirely on Day 28 (Fig 3B and 3E) showed similar histoarchitecture to normal skin (Fig 3A and 3D), with skin appendages had developed in the AB group. The ECM was loose identical to the normal skin, except the epidermis was slightly thicker than the normal epidermis. On the other hand, the NHI group showed hyper-epithelisation and compacted ECM; granulation tissue was massive with immense mononuclear cells scattered at the centre of the wound, indicating that the inflammation was still active, while skin appendages were scarce (Fig 3C and 3F).

IHC labelling against α-SMA in the AB group (Fig 3H) was found around the glands as seen in the normal section (Fig 3G); nonetheless, the ECM was more dominant than the normal sections. In contrast, the NHI group (Fig 3I) expressed less α-SMA compared to the normal sections, with less expression found in the ECM. The NHI group had a thick epidermal layer, with the dermis was crowded with inflammatory cells.

The collagen-1 labelling in the AB group (Fig 3K) was homogeneously positive across the sections, similar to the normal section (Fig 3J), while the NHI was only found in the deep layer (Fig 3L). The fibre direction in the AB group was also arranged according to the normal skin. The vWF labelling of the AB section was found dispersed intensely near the epidermis (Fig 3N), compared to the NHI group (Fig 3O). Whilst the MIP-3 α labelling was massive in the AB group (Fig 3Q) compared to the NHI group (Fig 3R), although the normal rat skin showed no staining of MIP-3 α (Fig 3P).

The gene expressions of the wound scar after 28 days experiment (7 days dressing and 21 days graft replacement) showed that the pro-inflammatory genes of the NHI group were highly expressed very significantly compared to the AB group, also increased significantly from Day 0 (Fig 1E).

## Discussion

A rat skin has a substantial skin layer similar to humans; however, the mechanism of wounds healing in rats by contraction, while humans through epithelialisation that heals slower [30, 31]. Even so, rats are common for use as a burn model, as they are easy to maintain, economical, and the wound heals fast [26]. Nonetheless, the skin cycle in rats appears heterogeneously. Therefore, it was homogenised by removing the hair at the telogen phase using wax to enter a similar skin cycle phase at the anagen phase [32, 33]. In this study, no additional analgesia was applied after the intervention to intervene in the wound healing process [34].

An eschar was formed after burn induction immediately. A study reported that wound healing up to three weeks increased scar formation up to 33% [35]. Therefore, skin transplantation is required for a deep thickness burn, minimising skin damages, and hoping that burn wounds will be fully recovered once skin transplantation is applied. The conditions happen especially in rural places, for example, when skin transplantation is unavailable for a burn victim. In this study, AB and Sofra-Tulle ^®^, were observed to apply as a dressing.

Our study showed that the application of AB immediately after burn for seven days improved the damage severity due to heat and promoted the restoration of skin appendages, compared to the standard protocol. The rats in the AB group appeared to be calm. After the treatment, able to maintain normal activities such as eating, urinating, and grooming. Reports indicated that pain causes animal labs to be aggressive [36, 37]. Initial days after burn induction, the rats from the NHI group mostly stood still but were very aggressive when weighed and the wound were touched unintentionally.

In contrast, the AB group rats were calm and responded similarly throughout the experiment while weighing. Although both groups did not differ significantly, except the weight of rats in the AB group after Day 7 until 14 was slightly higher, we assumed AB application might reduce pain compared to the NHI group. The wound healed significantly faster in the AB group by days 10 and 14. These happened once the scar was removed and replaced by a graft (AB vs Sofra-Tulle ^®^). The contraction rate in the wound healing process in rats is influenced by several factors, including age, treatment and depth of burn wound.

The sections of the AB group showed the recovery of collagen denaturation was prominent compared to the NHI group, which remained very red compared to the AB group. Collagen fibre has a cationic group and is denatured by heat and changes the configuration of these molecules that retained the Ponceau/Acid Fuchsin component from the Masson dyes, therefore appeared as dark pink or red. Dry heat, especially when it reached 100°C caused uniformly red, while in a moist environment, the direction of the fibres is still retained [38]. Masson staining can identify collagen denaturation grading. Which usually appears blue-green but becomes red when denaturated [39]. Moreover, the NHI group showed a massive granulation tissue in the deeper layer. After burning, the normal fibre collagen forms an amorphous globular material collagen structure that is more severely disorganised by gelatinisation skin [38].

Once the eschars were removed and grafted accordingly to each group, newly generated collagen fibres were formed in the AB group. The fibres’ direction was the same as the normal skin and had a similar loosened type of ECM. Fibre direction indicates collagen maturity and prevention for scar formation [19, 40].

After seven days of burn induction and dressing, the NHI group’s macrophage inflammatory protein-3 α (MIP-3-α) was strongly expressed. A superficial layer fully populated with mononuclear cells contrasts with the AB group. MIP-3 α cells are recruited, especially to an infected area and also found in a prolonged inflammatory response. These can disrupt the normal progression of wound healing [41]. According to Zhang et al. (2007), activated macrophages release MIP-3-α that is related to pathological pain [12]. A study reported that MIP-3α was highly expressed when a full-thickness burn rat after Day 3 and Day 7 was infected with *Pseudomonas aeruginosa* [42]. *P*. *aeruginosa* (a gram-negative bacterium) is the most common pathogen observed in burn patients, unresponsive to antibiotic treatments as it forms biofilms on wounds. Nonetheless, an acute stage of this infection results in an overactive immune response that induces a significant inflammation response [43–46]. In our study, the NHI group indicated massive mononuclear cells after seven days of dressing, with up to 30% cells were MIP-3α positive. The AB group also showed positive cells with MIP-3α; except, the cells were fewer.

Our study was terminated once the rats from any group showed to heal completely. After 28 days, no infection was observed in the AB group, while the wound in the NHI group remained active at the same time point. The infection was not observed until Day 28 (Fig 2). The AB has an amnion membrane, which naturally has an antimicrobial effect, especially against *Streptococcus* group A, *Pseudomonas aeruginosa* [47]. Antimicrobial peptides and β-defensin-1 found at the epithelial layer of the AB membrane can attach to the wound and reduce pain [48]. The decellularised biological graft had been reported to exhibit an antimicrobial property [49]. The centre of the eschar found in the NHI group after 28 days remained wet, indicating that the inflammation was still active. Further bacterial analysis was not performed for this time point; therefore, any infection activities was undetermined.

There are several markers to identify wound healing and scar formation. Immunohistochemistry labelling against collagen-1 indicates mature fibre. The α-SMA marks myofibroblastic in the early scar process but also the smooth muscles around blood vessels. Sections with α-SMA and vWF positive indicate angiogenesis and functional endothelial. During wound healing, fibroblasts will transform into myofibroblasts, as indicated by α-SMA. Myofibroblasts have a strong contractile force to retract the wound size [10]. The recruited fibroblast also can stimulate collagen deposition [50, 51].

In our study, the α-SMA expression was distributed evenly in the dermis, while the NHI group had less. Perhaps, the implantation of AB graft also recruited mesenchymal stromal cells to the injured site and induced angiogenesis. Angiogenesis is indicated by α-SMA expression identified from the smooth muscle in the wall of the newly formed blood vessels and the vWF labelling the endothelial. Our study showed that both α-SMA and vWF were expressed in the AB group, whilst the NHI group was also found. Report on decellularised hAM, overlaid with electrospun nanofibrous silk fibroin, stimulated neovascularisation and re-epithelialisation [19]. Moreover, our previous study showed that fibrin matrix cultured with human bone marrow mesenchymal stem cells generated high VEGF (up to 1300 pg/mL) after ten days of incubation (unpublished data). VEGF was important for regulating angiogenesis and neovascularisation by acting on endothelial cells [28].

Next, the epidermis of the NHI group had been thickened, compared to the normal skin or the AB group, while the collagen deposition was irregular. These might indicate a prolonged inflammatory response [41].

Pro-inflammatory cytokines such as IL-6 and TNF- α were reported to be increased during the early wound to initiate the healing response [52], usually by the day 0 to three after burning injury [12]. The activated macrophages mainly produce Pro-inflammatory cytokines such as IL-1β, IL-6, TNF-α, MIP-3 α, involved in the pathological pain [12]. Elevation of IL-6 initiates a healing response, while chronic wounds had persistently high expression [52]. The level of TNF-α (Tumor necrosis factor-α) expressions depends on the duration and degree of injury, which induces FGF-7 expression that promotes re-epithelialisation [53]. Nonetheless, the expression of TNF-α alone inhibits wound re-epithelialisation. In contrast, a low level of TNF-α expression stimulates inflammation by inducing the macrophage to produce growth factors and speed up wound healing. A persistent and high level of TNF-α is damaging to a wound healing process. It impedes ECM production and TIMPs, also increases MMPs (matrix metalloproteinases) synthesis (MMP-1, MMP-2, MMP-3, MMP-9, MMP-13, and MT1-MMP) [54–57]. Up-regulation of MMP activity hinders the healing process, as it degrades the ECM and halts cellular migration and collagen deposition. MMPs also impairs the growth factors and their target cell receptors [58].

In this study, the gene expressions of the pro and anti-inflammatory markers of NHI and AB groups wounds. The pro-inflammatory gene expressions increased significantly in the NHI group; TNF-α by 7-fold, IL-6 by 5.5-fold, and MMP-1 was up to 9-fold (Fig 1). The report indicated that increasing these markers is likely to form scars [59]. High concentrations of pro-inflammatory cytokines increase oxidative stress. They down-regulate eNOS (endothelial nitric oxide synthase) bioactivity and induce endothelial cell apoptosis [60].

Others showed that burn had a significant increase of IL-1α, GM-CSF, TNF, IL-6, IL-17, G- CSF, KC, MIP-1α, and RANTES expressions. The anti-inflammatory cytokines (IL-10, IFN-α, and IL-12p70) were initially decreased within the first 9 hours, then increased after 24 hours, followed by the normal range by day 10. The burn and sham mice showed no difference in expressing IL-1-α, IL-12p40, whilst the IL-4 was undetectable [61]. This report aligned with our study that the IL-10 was still high; after 21 days of transplantation or the Day 28 experiment. Anti-inflammatory cytokines are the immunoregulatory molecules that control the response of the pro-inflammatory cytokines. IL-10 is a robust anti-inflammatory cytokine that regulates the expression of TNF-α, IL-6 and IL-1 through the macrophages [12, 62]. Our study demonstrated that AB application after seven days of dressing and transplantation induced IL-10 expression up to 6-fold, significantly higher than the NHI group. In contrast, the NHI had no different to the sham.

Seok et al. (2013) analysed the relationship between gene expressions between humans and rats, focusing on a burning study. Pearson correlations (R2) of 40 most dominant pathways and suppressing after burn-in rats and humans; indicated that the percentage of genes and median correlations developed to the same direction were 67% and 0.16, respectively. The gene response time in both humans and rodents started by the first 6–12 h; however, the healing duration differed enormously. The rodents’ genes were stabilised by the first four days; in contrast, humans needed at least six months [63]. On the contrary, others report the IL-1β, IL-6, IL-10, monocyte chemotactic protein 1, CINC-1, CINC-2, and CINC-3 were increased significantly higher compared to the sham rats (p< 0.05), but the TNF-α and VEGF (vascular endothelial growth factor) were not different compared [64].

The AB group showed complete healing after 28 days of experiment or 21 days post-transplantation. The fibrin might contribute to this healing capacity in the amnion bilayer, generated from platelets rich plasma (PRP). PRP contained rich in growth factors such as fibronectin, vascular endothelial growth factor (VEGF), insulin growth factor (IGF-I) and interleukin -1b (IL-1b) [65]. Others reported enriched with platelet growth factor (PDGF), transforming growth factor-beta (TGF-β1), vascular endothelial growth factor (VEGF), and basic fibroblast growth factor (bFGF) [66] that support the healing rate shown by the AB group. These cytokines directly influence endothelial cell behaviours, such as proliferation, migration, specialisation, or just survival [67].

Wound healing processes are a cascade of inflammation, proliferation, and re-epithelialisation/remodelling [50, 51]. Inflammation is the initial response, and in this stage, inflammatory cells reduce cell debris or germs by a rigorous assimilation process [51]. PDGF accelerated the proliferation of fibroblasts and stimulated lattice re-epithelialisation and skin barrier function restoration [47, 68]. A study using decellularised human placenta-sheeted ECM (ECM sheets) on a full-thickness burn rat indicated that partial epithelialisation was seen after 14 days of implantation, as epidermal appendages were partially formed. TGF-β1 is believed to be upregulated during the process [69]. Matrix of ECM sheets that contained those growth factors and contributed to skin structure regeneration in 28 days [69].

The healing rate of a full-thickness burn in neonatal rats was reported faster than the adulthood, as the neonatal rats took three days, while adults up to 30 days or longer [70]. The re-epithelialisation phase was started on days 1–3 in the neonate rat compared to 3–10 days in the adults. Planimetric-wound assessment of full-thickness burn treated with BMSC (bone-marrow stromal cells) showed that the burn surface was reduced to 68% of the original area at day 14 by epithelialisation from the wound margin and wound constriction. The presence of fibroblast-like cells increased in the neodermis and scar tissue between days 10 and 14 postburn. Regenerated epidermal keratinocytes migrated from the edge to the central area of the wound along with regenerated neodermis [71]. Adult rats treated with human placenta-derived extracellular matrix (ECM sheets) remodelled rapidly within 7–14 days. The healing scar was reported to recover with a continuous epidermis. Skin appendages had partially formed by day 14, and the basement membrane was well developed, the epidermis layers, suprabasal granular by day 28. Reepithelialization was reported to peak 10–14 days after injury [19, 70, 71].

Our study showed that statistical analysis of wound size was different significantly between NHI and AB groups. The report indicated that EGF (epidermal growth factor) promoted re-epithelialisation and wound closure processes [69]. Some adult rats with full-thickness burn fastest healed naturally after 30 days and might be longer. The scars indicated an irregular collagen deposition and incomplete-sparse skin appendages, forming a new thickened epidermis [70]. In our study, the wound site of the AB group fully recovered on Day 28, with newly formed epidermis was similar to the normal rat skin, with skin appendages such as sebaceous gland, hair follicle and other skin appendages were formed. The NHI group showed the thickened epidermal appearance described by Wagner et al. (2007) [70]. Our study was terminated after 28 days; nonetheless, the NHI group remained active in inflammation.

## Conclusions

In this study, a full-thickness burn was successfully modelled in rats and biological matrix application. In conditions when skin transplantation is delayed, AB as a dressing showed to be encouraging. AB application as a graft to replace the skin also shown to heal faster than the standard protocol when skin transplantation is not available with full recovery after 28 days, without a scar, and with skin appendages developed. The epidermis was slightly thicker than the normal skin but not as thick as the group without graft.

## Supporting information

S1 Table Rat body weight. (XLSX)

S2 Table Real-time qPCR raw data. (XLSX)
